# Functional metagenomics identifies an exosialidase with an inverting catalytic mechanism that defines a new glycoside hydrolase family (GH156)

**DOI:** 10.1074/jbc.RA118.003302

**Published:** 2018-09-24

**Authors:** Léa Chuzel, Mehul B. Ganatra, Erdmann Rapp, Bernard Henrissat, Christopher H. Taron

**Affiliations:** From ‡ New England Biolabs, Ipswich, Massachusetts 01938,; the § Max Planck Institute for Dynamics of Complex Technical Systems, 39106 Magdeburg, Germany,; ¶ glyXera GmbH, 39120 Magdeburg, Germany,; the ‖ Architecture et Fonction des Macromolécules Biologiques, CNRS, Aix-Marseille Université, F-13288 Marseille, France,; the ** Institut National de la Recherche Agronomique (INRA), Unité Sous Contrat (USC) 1408, Architecture et Fonction des Macromolécules Biologiques, 13288 Marseille, France, and; the ‡‡ Department of Biological Sciences, King Abdulaziz University, Jeddah 21589, Saudi Arabia

**Keywords:** sialidase, glycoside hydrolase, high-throughput screening (HTS), neuraminidase, sialic acid, glycosylation, extremophile, functional metagenomics, inverting mechanism

## Abstract

Exosialidases are glycoside hydrolases that remove a single terminal sialic acid residue from oligosaccharides. They are widely distributed in biology, having been found in prokaryotes, eukaryotes, and certain viruses. Most characterized prokaryotic sialidases are from organisms that are pathogenic or commensal with mammals. However, in this study, we used functional metagenomic screening to seek microbial sialidases encoded by environmental DNA isolated from an extreme ecological niche, a thermal spring. Using recombinant expression of potential exosialidase candidates and a fluorogenic sialidase substrate, we discovered an exosialidase having no homology to known sialidases. Phylogenetic analysis indicated that this protein is a member of a small family of bacterial proteins of previously unknown function. Proton NMR revealed that this enzyme functions via an inverting catalytic mechanism, a biochemical property that is distinct from those of known exosialidases. This unique inverting exosialidase defines a new CAZy glycoside hydrolase family we have designated GH156.

## Introduction

Enzymes are nature's biological catalysts. They perform chemical reactions with remarkable speed and specificity, making them critical for the biochemical processes that sustain life. Most enzymes have evolved to perform their function under moderate environmental conditions that support the majority of life on our planet. However, many organisms thrive in physically and chemically extreme environments such as hot springs, hydrothermal vents, hypersaline ponds, evaporation salterns, and acidic or soda lakes. These extremophiles often produce enzymes with biochemical properties that have been uniquely shaped by their extreme surroundings. As such, the study of enzymes from microbial communities that populate extreme environments is of interest to both basic science and biotechnology.

Over the past decade, functional metagenomics has become increasingly popular for identification of novel enzyme activities in nature. In a functional metagenomics workflow, high-molecular-weight DNA is extracted *en masse* from microbes that populate an environmental niche. This environmental DNA is cloned into a fosmid vector and introduced into a host cell, often *Escherichia coli* (1). Individual *E. coli* clones can then be screened in high throughput for expression of a desired enzyme activity using an appropriate reporter substrate. This approach is advantageous because it allows for enzyme discovery from both cultivable and uncultivable microorganisms from any environmental niche. Additionally, because the screening is based on enzyme activity and not sequence similarity to known enzymes, truly novel and unique proteins can be identified.

Functional metagenomics has been used to identify a variety of carbohydrate hydrolases (glycosidases) from environmental libraries (2–4). Several studies have reported discovery of novel glycosidases (cellulases, glucosidases, xylanases, etc.) that improve hydrolysis of plant polysaccharides with the aim of improving biomass conversion for biofuel production (3, 5–7). Additionally, novel glycosidases that improve food processing techniques have also been reported (8). Most of these studies have interrogated libraries from mesophilic microbial populations from intestinal, terrestrials or aquatic niches. Only a few studies have identified novel glycosidases from extremophiles, although cold, heat, and alkaline tolerant glycosidases have been reported (9–11). The majority of glycoside hydrolase functional metagenomics studies have sought enzymes involved in degradation of plant biomass, an expected ability of many microbial populations that occupy terrestrial niche. However, the diversity of glycosidase specificities present in extremophile populations remains largely unexplored. We have an interest in enzymes that degrade complex oligosaccharides found on eukaryotic glycoproteins and glycolipids. As such, we have begun screening various extremophile libraries for activities typically associated with complex glycan degradation (*e.g.* sialidases, galactosidases, fucosidases, mannosidases, etc.). In the present study, we conducted a functional metagenomic screen of an environmental DNA library from a freshwater thermal spring (average temperature, ∼60 °C) for enzymes capable of hydrolysis of sialic acids (“sialidases” or sometimes “neuraminidases”; EC 3.2.1.18).

Sialic acids are a structurally diverse family of monosaccharides that share a common nine-carbon backbone. In eukaryotes, they are generally found in a terminal position on *N*-glycans, *O*-glycans, glycosphingolipids, and glycosylphosphatidylinositol anchor side branches. Additionally, many bacteria that closely associate with animals incorporate sialic acids into their cell walls (12). In mammals, the most common form of sialic acid is modified at the C-5 position with an acetyl group (Neu5Ac). However, in nonhuman vertebrates a hydroxylated acetyl group (Neu5Gc) can occupy the C-5 position. Commonly, a sialic acid residue is attached to an adjacent neutral sugar (*e.g.* galactose, GalNAc, GlcNAc) via an α2–3 or α2–6 linkage. However, other linkages are also possible, including polysialic acid chains, in which many Neu5Ac residues are each connected via an α2–8 linkage (13).

Sialidases are enzymes that catalyze hydrolysis of the glycosidic bond that links a sialic acid to a subterminal sugar in an oligosaccharide. They are widely distributed in biology and have been found in animals, fungi, protozoa, bacteria, and many viruses. Based on their amino acid sequences, sialidases have been classified into four CAZy (Carbohydrate-Active Enzymes Database, www.cazy.org)^2^ hydrolase families (14). Families GH33, GH34, and GH83 each contain exosialidases, enzymes that release a terminal sialic acid from oligosaccharides. In addition, family GH58 comprises endosialidases, enzymes that cleave within a polysialic acid chain (15). Families GH34 and GH83 are exclusive to viral neuraminidases, whereas family GH33 contains all known bacterial and mammalian exosialidases. Despite their lack of primary amino acid sequence similarity, all four of these sialidase families share a common 6-fold β-propeller tertiary structure (16–23). Mechanistically, known exosialidases release sialic acid with overall retention of its anomeric conformation (24–27). In contrast, endosialidases function via an inverting catalytic mechanism (28).

In the present study, we report functional metagenomic screening of a freshwater thermal hot spring fosmid library for sialidase activity. To our knowledge, this is the first functional metagenomic survey aimed at identification of sialidase activity from an extreme environment. We report the discovery and biochemical characterization of a novel exosialidase that defines a new CAZy glycoside hydrolase family (GH156). Additionally, we show that this newly found enzyme catalyzes sialic acid hydrolysis via an inverting reaction mechanism, the first example of a WT exosialidase with this ability.

## Results

### Metagenomic DNA library construction and functional screening

**Figure 1.**
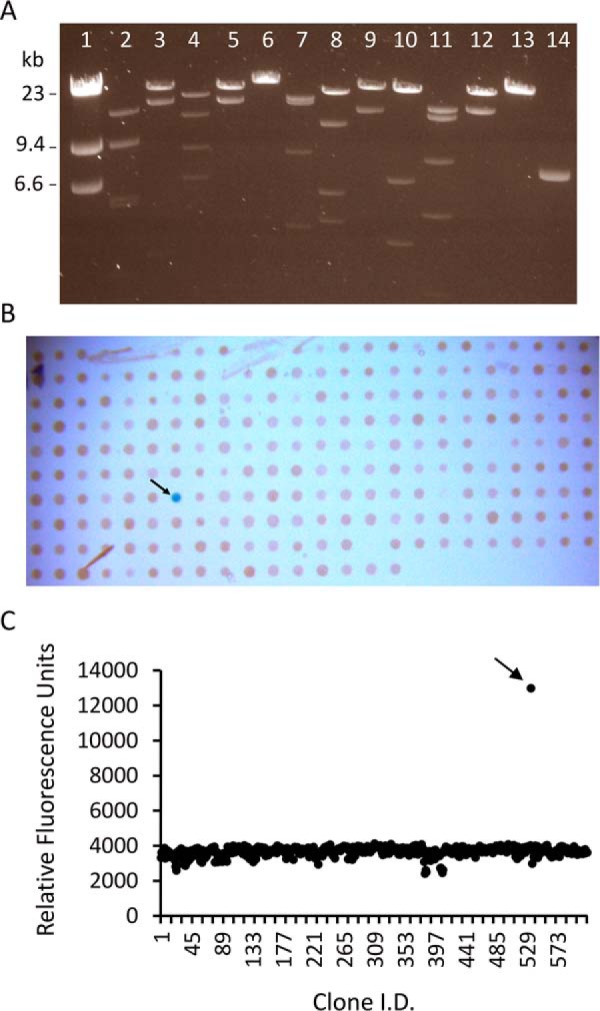
**Screening for sialidase activity from a hot spring metagenomic library.** *A*, restriction fragment analysis of 12 randomly selected clones from the hot spring metagenomic library were isolated and digested with the rare-cutting endonuclease SbfI. Digested fosmids were separated overnight on a 1% agarose gel along with a λHindIII size marker and a linearized pSMART FOS empty vector control (*lane 14*; contains one SbfI site). Each clone showed a unique banding pattern, with fragments whose combined sizes indicated the presence of an insert of at least 30–40 kb. *B*, *E. coli* cells harboring individual fosmid clones were assayed for sialidase activity with X-Neu5Ac incorporated into agar medium. A single positive clone forming a *blue* colony is denoted with an *arrow. C*, lysate from microcultures of *E. coli* cells harboring individual fosmid clones were assayed for sialidase activity with 4MU-α-Neu5Ac. A single positive clone is denoted with an *arrow*.

A small metagenomic DNA library was constructed from environmental DNA isolated from hot spring mats collected in Dixie Valley, Nevada. The library was introduced into *E. coli*, and 616 individual clones were arrayed into two 384-well plates to facilitate enzyme screening. Restriction fragment analysis of 12 randomly selected library clones indicated an average cloned insert size of ∼30–40 kb (Fig. 1*A*). Additionally, Sanger sequencing of the ends of each insert revealed that cloned DNA originated from both known and unknown bacterial species. Among the known species were the thermophiles *Caldilinea aerophila* and *Thermomicrobium roseum* (29, 30). Other clones were related to *Chloroflexus* sp., *Thermocrinis ruber*, or *Acidobacteria bacterium*, and five clones contained DNA from unknown origins. Thus, even in a small sampling of randomly isolated clones, the library harbored genetic material from a diverse range of microbes.

To identify active sialidases, the arrayed clones were screened with 5-bromo-4-chloro-3-indolyl α-d-*N*-acetylneuraminic acid (X-Neu5Ac)^3^ and 2′-(4-methylumbelliferyl)-α-d-*N*-acetylneuraminic acid (4MU-α-Neu5Ac) substrates in agar plate and cell lysate assays, respectively. In the agar plate screen, a single colony (designated G7) hydrolyzed X-Neu5Ac and turned blue after an overnight incubation at 37 °C and several days of incubation at 4**°**C (Fig. 1*B*). In the cell lysate screen, the same clone, G7, was identified by measuring an increase in fluorescence caused by the hydrolysis of 4MU-α-Neu5Ac (Fig. 1*C*). No additional clones gave rise to detectable sialidase activity.

### Sialidase gene identification

To identify the gene responsible for the observed sialidase activity, fosmid G7 was sequenced using the PacBio RS II platform. A total of 44,770 reads were generated with an average insert size of 7,523 bp and an average polymerase read length of 15,285 bp. The reads were assembled with the RS_HGAP_Assembly.3 algorithm (31). After removal of the repeat region (often observed during *de novo* assembly of circular sequences), a single contig corresponding to the full insert nucleotide sequence (41,198 bp) was obtained (GenBank^TM^ accession number MH016668). The sequence coverage was ∼8,000×. The fosmid insert did not match any DNA sequence in GenBank^TM^, indicating that it was from a previously unsequenced organism.

**Figure 2.**
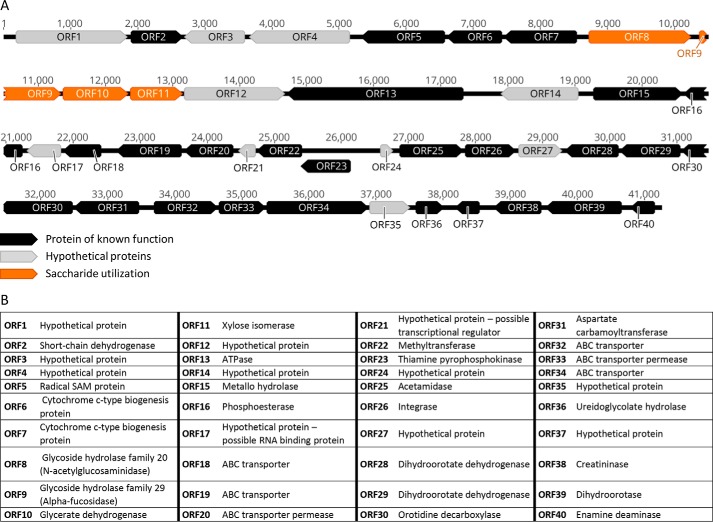
**Predicted ORFs encoded in the fosmid G7 nucleotide sequence.** *A*, fosmid G7 was sequenced on the PacBio RSII platform. ORFs were predicted with MetaGeneMark and classified into three categories based on their homology to proteins from the nonredundant protein database (NCBI). *Black*, proteins with a known annotated function; *gray*, “hypothetical proteins” with no annotated function; *orange*, proteins involved in saccharide utilization. *B*, database annotations for all 40 ORFs.

The fosmid G7 DNA sequence encoded 40 putative ORFs that were predicted by MetaGeneMark (32). BLASTP analysis of the nonredundant GenBank^TM^ protein sequence database was performed using each predicted ORF protein sequence as a query. Based on the observed similarity of each ORF to homologous proteins in GenBank^TM^, the 40 ORFs were classified into three categories: (i) proteins of known function, (ii) proteins involved in saccharide utilization, and (iii) hypothetical proteins of unknown function (Fig. 2, *A* and *B*). Of the 40 ORFs, four showed similarity to proteins involved in saccharide utilization, and none showed similarity to proteins from the known CAZy sialidase families GH33, GH34, GH58, and GH83.

**Figure 3.**
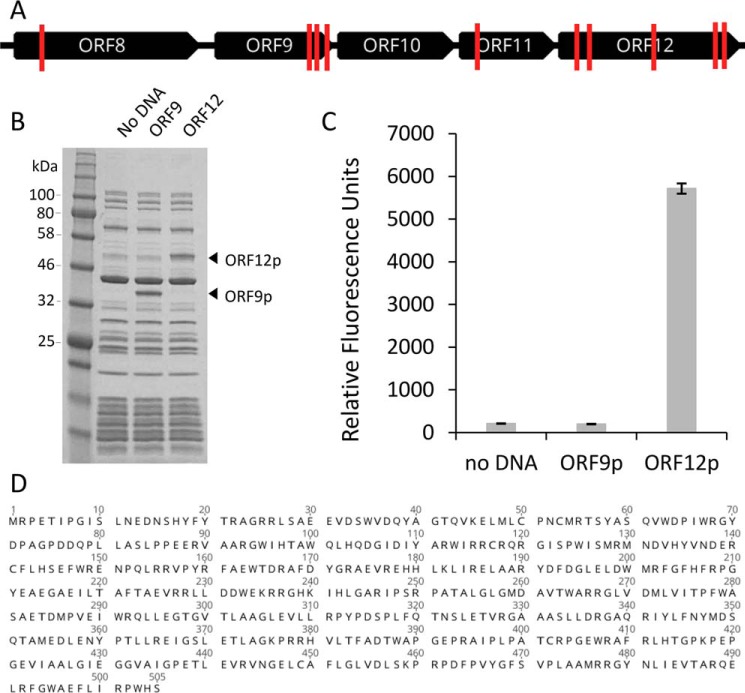
**Identification of the sialidase-encoding ORF on fosmid G7 and its *in vitro* expression.** *A*, a map of fosmid G7 transposon insertion sites (*red lines*) in mutants with abolished sialidase activity. *B*, SDS–PAGE of ORF9 and ORF12 proteins expressed *in vitro* using the PURExpress system. *C*, sialidase activity produced in PURExpress reaction mixtures was assessed using the substrate 4MU-α-Neu5Ac as described under “Experimental procedures.” *D*, the deduced amino acid sequence of ORF12p. The nucleotide sequence and the deduced protein sequence for ORF12 are annotated in the fosmid G7 sequence record (GenBank^TM^ accession number MH016668).

Because sequence analysis did not identify an obvious sialidase candidate among the 40 sequenced ORFs, transposon mutagenesis was performed to disrupt expression of the sialidase activity. Tn5 mutagenesis was used to randomly insert a kanamycin cassette into fosmid G7 under conditions that minimized multiple insertion events. A total of 192 mutants were assayed for sialidase activity using the substrate 4MU-α-Neu5Ac (Fig. S1). Seventeen mutants with abolished sialidase activity were identified. Each of these mutants was bidirectionally Sanger-sequenced with transposon-specific primers to identify the element's insertion site. Multiple transposon insertion events were observed for seven mutants, and they were not further investigated. A map of the G7 insertion sites for the remaining ten mutants is shown in Fig. 3*A*. All loss-of-function insertions clustered to a ∼5-kb region of fosmid G7 containing several ORFs with similarity to enzymes that hydrolyze or metabolize sugars (Figs. 2*A* and 3*A*). The majority of insertions occurred in ORF9 (annotated as encoding a putative α-l-fucosidase) or ORF12 (annotated as encoding a “protein of unknown function”).

To determine whether either ORF9 or ORF12 encoded a protein with sialidase activity, each was expressed using the PURExpress *in vitro* protein synthesis system (Fig. 3*B*) and tested for its ability to cleave 4MU-α-Neu5Ac (Fig. 3*C*). The expressed product from ORF12 (ORF12p) generated a fluorescence signal ∼27 times higher than the no DNA control reaction (the PURExpress mix alone). Expression of ORF9 yielded no sialidase activity. Because the deduced protein sequence of ORF9 (ORF9p) had sequence homology to α-l-fucosidases, the ability of *in vitro* expressed ORF9p to cleave the chromogenic fucosidase substrate 2-chloro-4-nitrophenyl α-l-fucopyranoside was also tested. Light absorbance at 405 nm was approximately six times higher for ORF9p than the negative control, confirming that the ORF9 encoded an α-fucosidase, not an exosialidase. Together, these data support the conclusion that the gene product from ORF12 (Fig. 3*D*; GenBank^TM^ accession number MH016668) is solely responsible for the hydrolysis of 4MU-α-Neu5Ac observed during primary screening. Furthermore, because ORFs 8–12 are co-directionally oriented and are each related in some way to saccharide metabolism, it is plausible that these genes are co-transcribed in the organism from which the locus originates and in *E. coli*. This could account for the observation of reduced sialidase activity upon insertion of Tn5 into multiple positions in this region.

### Sialidase biochemical characterization

**Figure 4.**
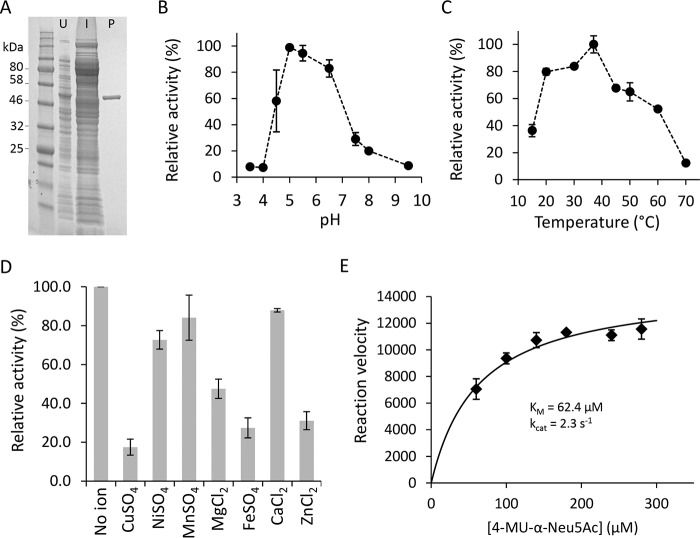
**Purification and biochemical characterization of recombinant ORF12p.** *A*, His-tagged ORF12p sialidase was expressed in *E. coli* and purified using a His-trap column as described under “Experimental procedures.” Shown is SDS–PAGE separation of lysates from uninduced cells (*U*), induced cells (*I*), and nickel-purified ORF12p–His (*P*). *B–D*, purified ORF12p–His was used to determine the pH (*B*) and temperature (*C*) optima of ORF12p–His and the effect of metal ions on its catalysis (*D*). In these experiments, reactions were performed in triplicate using the substrate 3′-sialyl-*N*-acetyllactosamine-2AB. Reaction products were analyzed by UPLC–HILIC–FLR and quantitated by peak integration. *E*, Michaelis–Menten plot of ORF12p catalyzed hydrolysis of 4MU-α-Neu5Ac. The initial velocity was determined in triplicate for each 4MU-α-Neu5Ac concentration.

To facilitate biochemical characterization of ORF12p, an expression DNA construct encoding ORF12p with a C-terminal hexahistidine tag (ORF12p–His) was assembled. ORF12p–His was expressed in *E. coli* and purified using nickel-affinity chromatography (Fig. 4*A*).

The substrate 3′-SLN-2AB was used to determine the optimum pH and temperature of the ORF12 sialidase as described under “Experimental procedures.” A pH optimum of 5.0–5.5 was observed (Fig. 4*B*). The enzyme showed optimal activity at 37 °C and retained at least 50% activity at 60 °C (Fig. 4*C*). No metal ions were required for activity (Fig. 4*D*). Reaction kinetics were determined using the substrate 4MU-α-Neu5Ac. The initial reaction rates were determined at several substrate concentrations (Fig. 4*E*). The *K_m_* was calculated to be 62 μm, and the *k*_cat_ was calculated to be 2.3 s^−1^.

**Figure 5.**
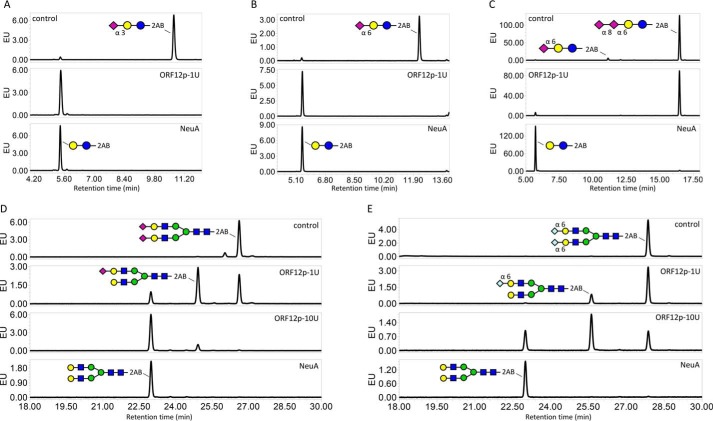
**Specificity of ORF12p on sialic acid containing substrates using UPLC–HILIC–FLR analysis.** *A* and *B*, the ability of ORF12p to cleave the fluorescently labeled substrates 3′- or 6′-sialyl-*N*-acetyllactosamine-2AB. Undigested substrates 3′- or 6′-sialyllactosamine-2AB run at ∼10.6- or 12.3-min retention times, respectively (*A* and *B*, *top panels*). Control digestion with the NeuA sialidase shifted both substrate peaks to ∼5.5-min retention time (*A* and *B*, *bottom panels*). Digestion of these substrates with 1 unit of ORF12p–His resulted in the same peak shift (*A* and *B*, *middle panels*). *C*, ORF12p's ability to hydrolyze α2–8 Neu5Ac was assessed using a 2AB-labeled GD3 ganglioside headgroup substrate that contains two sialic acid residues linked via an α2–8 bond. Undigested substrate ran at ∼16.5-min retention time with a very minor peak at ∼11-min retention time corresponding to partially degraded substrate comprised of a single α2–6 terminal sialic acid (*C*, *top panel*). NeuA-treated substrate shifted at ∼5.5 min retention time (*C*, *bottom panel*). Treatment with 1 unit of ORF12p did not shift the major substrate peak (*C*, *middle panel*). *D* and *E*, activity of ORF12p on biantennary complex *N*-glycans with terminal sialic acid residues (Neu5Ac, *D*; or Neu5Gc, *E*). Undigested substrates run at ∼26.6- and 27.9-min retention time, respectively (*D* and *E*, *top panels*). NeuA treatment shifted both substrate peaks at ∼23-min retention time (*D* and *E*, *bottom panels*). Incubation of the substrates with 1 or 10 units of ORF12p resulted in the same peak shift, but incomplete substrate desialylation was observed resulting in another smaller peak shift at ∼24.9- and 25.6-min retention time, respectively (*D* and *E*, *middle panels*). Symbolic representation of glycan structures was drawn following the guidelines of the Consortium for Functional Glycomics (50). *EU*, emission units.

The specificity of purified ORF12p–His was assayed using 2-aminobenzamide–labeled 3′- and 6′-sialyl-*N*-acetyllactosamine substrates (termed 3′-SLN-2AB and 6′-SLN-2AB, respectively), a GD3 ganglioside headgroup glycan (Neu5Ac(α2–8)Neu5Ac(α2–3)Gal(β1–4)Glc-2AB) and G2S2 biantennary *N*-glycans with two terminal Neu5Gc or Neu5Ac residues (G2S2-Ac or G2S2-Gc). Digestion products were analyzed by ultra-HPLC with fluorescence detection (UPLC–HILIC–FLR) (Fig. 5). Negative control reactions consisting of each substrate and no enzyme yielded a single major peak for all five substrates (Fig. 5, *A–E*, *top panels*). A positive control reaction using commercial α2–3,6,8,9-neuraminidase A (NeuA; New England Biolabs) resulted in a shift of each substrate, illustrating complete removal of α2–3–, α2–6–, and α2–8–terminal sialic acid (Fig. 5, *A–E*, *bottom panels*). This same peak shift was observed for 3′-SNL-2AB and 6′-SNL-25AB substrates when incubated with purified ORF12p–His, indicating that this enzyme was able to hydrolyze both terminal α2–3– and α2–6–Neu5Ac (Fig. 5, *A* and *B*, *middle panels*) and confirming the ability of ORF12p–His to function as an exosialidase. The peak corresponding to the released glycan from GD3 ganglioside did not shift upon incubation with ORF12p–His, confirming the enzyme's inability to hydrolyze α2–8–linked sialic acid (Fig. 5*C*, *middle panel*). *N*-Glycan substrates G2S2-Ac and G2S2-Gc were both hydrolyzed by ORF12p–His, indicating that this enzyme can hydrolyze both terminal Neu5Ac and Neu5Gc from complex *N*-glycan structures (Fig. 5, *D* and *E*, *middle panels*). However, the enzyme showed a preference for Neu5Ac. In addition, the enzyme was assayed on a 2AB-labeled *O*-glycan library (Fig. S2) and showed the ability to hydrolyze terminal sialic acid linked to GalNAc or galactose residues.

### ORF12p sialidase reaction mechanism

Glycoside hydrolases are grouped into two mechanistic classes: inverting or retaining enzymes, depending on whether the stereochemistry of the substrate's anomeric carbon is inverted or retained upon hydrolysis. To date, known exosialidases function as retaining glycoside hydrolases, because they hydrolyze terminal Neu5Ac with net retention of its overall anomeric configuration (24–27).

**Figure 6.**
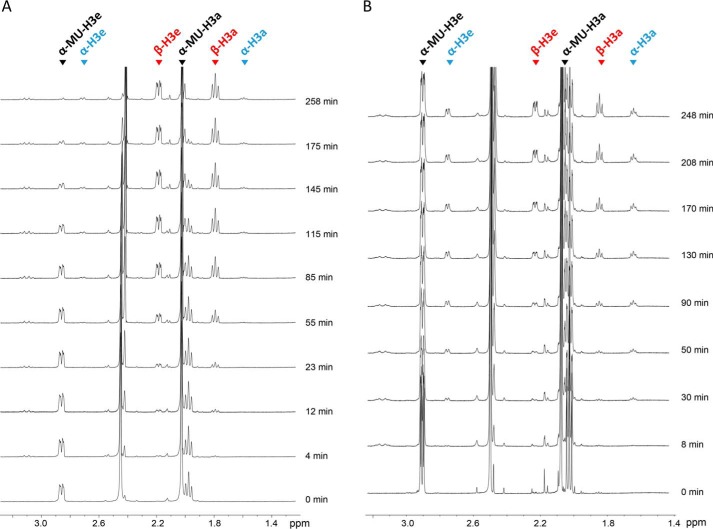
**Stereochemical course of the hydrolysis reaction catalyzed by ORF12p (*A*) compared with NeuA (*B*).** One-dimensional proton NMR was used to monitor the reaction products formed over time upon hydrolysis of 4MU-α-Neu5Ac by ORF12p and NeuA. *A*, the up-field region of the NMR spectra showed two groups of peaks at 1.99 and 2.86 ppm corresponding to the substrate 4MU-α-Neu5Ac (*black triangles*). Over time, groups of peaks appeared at 1.81 and 2.20 ppm corresponding to released β-Neu5Ac (*red triangles*). After 1 h, two sets of signals at 1.61 and 2.72 ppm appear as a result of spontaneous mutarotation of β-Neu5Ac to α-Neu5Ac (*blue triangles*). *B*, the spectra showed the substrate 4MU-α-Neu5Ac groups of peaks at 2.03 and 2.89 ppm (*black triangles*). After 30 min, H3a and H3e peaks corresponding to the α-anomer appeared at 1.64 and 2.76 ppm (*blue triangles*). These were quickly converted by mutarotation to the β-anomer at 1.85 and 2.23 ppm (*red triangles*).

Proton NMR (^1^H NMR) spectroscopy was used to determine whether ORF12p functions via a retaining or inverting mechanism. In this experiment, purified ORF12p–His was incubated with the 4MU-α-Neu5Ac substrate. Data analysis focused on the up-field region of the NMR spectra, where signals from the axial and equatorial protons in the 3-position of Neu5Ac appear. The initial NMR spectrum (Fig. 6*A*) showed only the axial (α-MU-H3a) and equatorial (α-MU-H3e) protons from the substrate 4MU-α-Neu5Ac at 1.99 and 2.86 ppm, respectively. The first additional set of signals (H3a and H3e) appeared after only a few minutes at 1.81 and 2.20 ppm. These signals corresponded to β-Neu5Ac that had been released from the substrate by the enzyme. The signal set of H3a and H3e corresponding to α-Neu5Ac (at 1.61 and 2.72 ppm) appeared after ∼1 h and remained very small throughout the remaining reaction. This reflects spontaneous mutarotation of the β-Neu5Ac product to its α conformer and is not a result of the action of the sialidase. These data unambiguously show that the ORF12 sialidase initially produces β-NeuAc from 4MU-α-Neu5Ac and supports the conclusion that the enzyme functions via an inverting mechanism.

A control experiment was run with purified NeuA (New England Biolabs), a known retaining exosialidase from the GH33 family (33). The initial NMR spectrum (Fig. 6*B*) showed the axial (H3a) and equatorial (H3e) protons corresponding to 4MU-α-Neu5Ac at 2.03 and 2.89 ppm, respectively. Upon initiation of the reaction, the first additional signals appeared after ∼30 min and corresponded to the α-anomer (1.64 and 2.76 ppm). These peaks were initially larger than the H3a and H3e β-Neu5Ac peaks (1.85 and 2.03 ppm, respectively). However, after 130 min, the peaks of the β-anomer became more prominent than those of the α-anomer, consistent with the spontaneous mutarotation of the α-Neu5Ac product to its β-Neu5Ac anomer. This was previously reported to reach an equilibrium mixture of 92.1% of β-anomer and 7.5% of α-anomer and three open-chain species at pH 8.0 (34). This stereochemical pattern of product release is characteristic of retaining exosialidases and is markedly different from that of ORF12p–His, further supporting our conclusion that ORF12p–His functions via an inverting mechanism of hydrolysis.

### ORF12p sialidase family phylogeny

**Figure 7.**
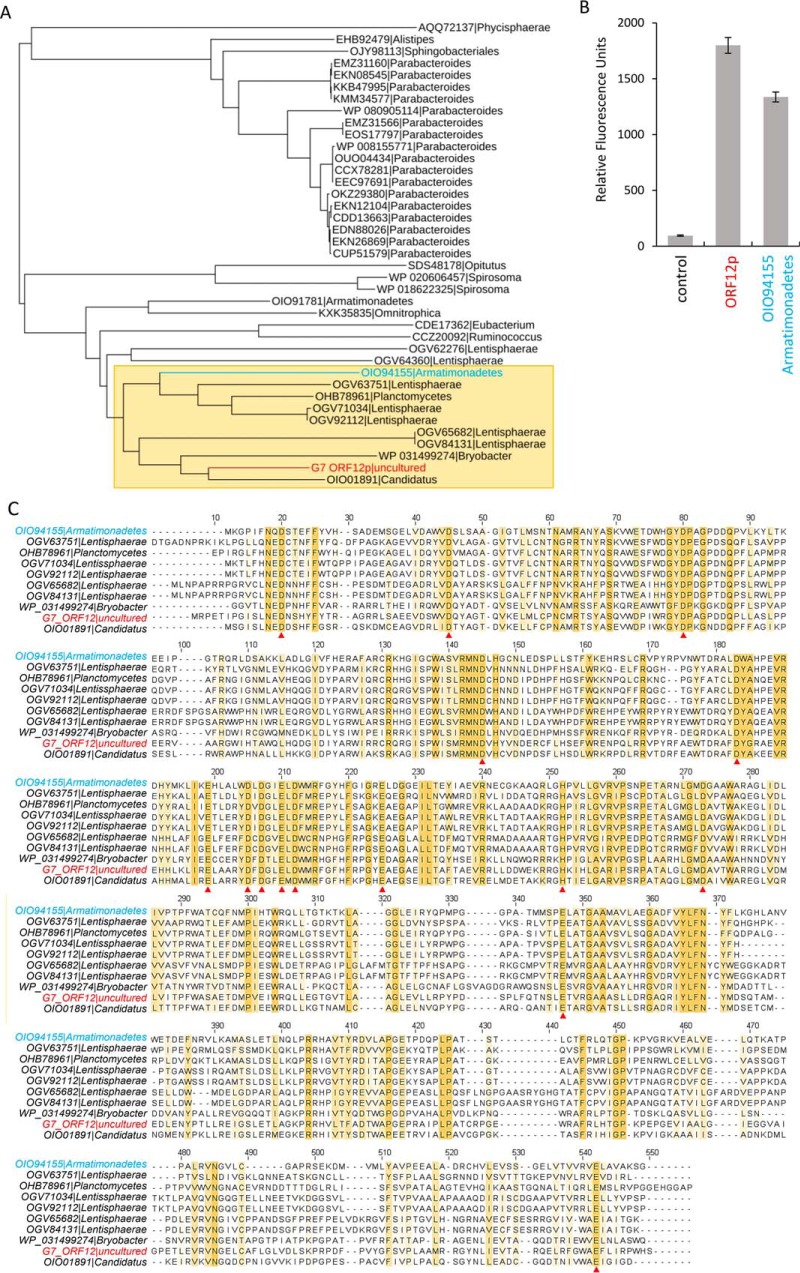
**ORF12 protein family.** *A*, ORF12p homologs were identified with the BLASTP algorithm and aligned with Muscle. A phylogenetic tree was then generated with the BLOSSUM62 matrix and the neighbor-joining method. ORF12p and *Armatimonadetes* OIO94155 are shown in *red* and *blue text*, respectively. *B*, the *Armatimonadetes* OIO94155 protein homolog was expressed in *E. coli* and crude lysate was tested with 4MU-α-Neu5Ac. *C*, protein sequence alignment (Muscle) of ORF12p and its closest homologs (*A*, *yellow box*), including *Armatimonadetes* OIO94155. Conserved residues are highlighted in *yellow*, and those potentially involved in catalysis are shown with *red triangles*.

ORF12p homologs were identified in the GenBank^TM^ sequence repository using the BLASTP algorithm. The 38 nearest homologs of ORF12 were retrieved and used for study of ORF12p phylogeny. Signal peptides that were present in many sequences were trimmed along with C-terminal extensions present for two homologs (OGV64360 Lentisphaerae and KXK35835 Omnitrophica). Trimmed sequences ranging from 477 to 578 amino acids were then aligned with Muscle, and a phylogenetic tree was made using BLOSSUM62 substitution matrix and the neighbor-joining method (Fig. 7*A*). It is noteworthy that all 38 ORF12p homologs showed no similarity to known CAZy glycoside hydrolases or other proteins of known function, and all were annotated as hypothetical proteins.

All sequences comprising the ORF12p family originated from bacteria and were distributed within eight prokaryotic phyla. The majority of sequences originated from organisms in the phylum Bacteroidetes or the superphylum Planctomycetes, Verrucomicrobia, and Chlamydiae (PVC) that contains the phyla Planctomycetes, Verrumicrobia, Omnitrophica, and Lentisphaerae. Other sequences were from organisms from the phyla Firmicutes, Armatimonadetes, and Acidobacteria. Based on our phylogenetic analysis, ORF12p was most closely related to sequences from organisms of the PVC superphylum. Notably, no homolog was found within the phylum Proteobacteria, which contains most of the well-studied bacteria, or in the phyla Actinobacteria or Cyanobacteria, which contain many species widely represented in soil and aquatic environments. Similarly, no eukaryotic or archaeal proteins had similarity to ORF12p.

To determine whether a second member of the ORF12p sequence family also had sialidase activity, the homologous protein OIO94155 from *Armatimonadetes* was expressed in *E. coli*, and crude lysate was assayed with 4MU-α-Neu5Ac. Lysate from *E. coli* expressing ORF12p was also assayed. A high fluorescence signal was detected for both ORF12p and OIO94155 lysates, indicating that OIO94155 is also an exosialidase (Fig. 7*B*). A multiple sequence alignment of 10 proteins (including ORF12p and OIO94155) showed the conserved regions within this family (Fig. 7, *A*, *yellow box*, and *C*). Strictly conserved aspartate, glutamate, and histidine residues are indicated (Fig. 7*C*, *red triangles*). These types of residues are commonly involved in catalysis in most inverting glycoside hydrolases (35–39). Thus, these positions will be candidates for future site-directed mutagenesis studies.

The cloned fosmid DNA sequence encoding ORF12p was unique and showed no significant match to DNA sequences from known organisms, thus providing no clue to its precise origin. The gene encoding ORF12p resided in a gene cluster that also encoded a GH29 family α-fucosidase and a GH20 family *N*-acetylglucosaminidase (Fig. 2). Although this locus did not conform to the currently defined structural features of a polysaccharide utilization locus (40, 41), the proximity of these genes and broad specificity of ORF12p suggests that these proteins may function in a coordinated manner, perhaps in degradation of eukaryotic complex *N*-glycans that at times could be present in a hot spring (*i.e.* decaying animal or plant matter).

## Discussion

In the present study, we utilized functional metagenomic screening to search for environmental enzymes with the ability to hydrolyze the fluorogenic sialidase substrate 4MU-α-Neu5Ac. Our screen identified a 41-kb environmental DNA fragment from a hot spring metagenomic library that produced sialidase activity in *E. coli*. The DNA fragment originated from an unknown microorganism and encoded no ORFs with homology to known sialidase families. Tn5 mutagenesis was used to identify a 505-amino acid ORF responsible for the observed sialidase activity (ORF12). We undertook the phylogenetic analysis of the ORF12 protein (ORF12p) family and biochemical characterization of this new sialidase.

Glycoside hydrolases and other enzymes that assemble or modify oligo- and polysaccharides are classified into families based on their amino acid sequence similarity (14). These enzymes are catalogued by family in the curated CAZy database. Currently, there are three distinct CAZy families of exosialidases; GH33, GH34, and GH83. The ORF12p sialidase identified in this study showed no homology to any members of these exosialidase families or to members of GH58, a family of endosialidases. Searches of public sequence repositories revealed that ORF12p was a member of a small family of proteins that are distributed only among bacteria from approximately eight phyla, several of which have been found in aquatic environments. No ORF12p family members were found in the most common bacterial phyla (*i.e.* Proteobacteria), archaea, or eukaryotes. Thus, this new sialidase family appears to be highly niche-specific in bacterial biology. The ORF12 protein sequence family defines a novel CAZy glycoside hydrolase family that has been designated GH156.

Biochemical characterization of ORF12p revealed both functional similarities and differences with known sialidases. ORF12p was capable of removing α(2,3)- and α(2,6)-Neu5Ac from the terminal position of oligosaccharides, indicating that it functions as an exosialidase. Despite lacking protein sequence similarity with known exosialidases, ORF12p shares similar basic biochemical properties with them (42–44). For example, ORF12p was broadly active from pH 4.5 to 8.0 (optimal at pH 5.0) and from 20 to 60 °C (optimal at 37 °C) and did not require metal ions, all features consistent with enzymes from the GH33, GH34, and GH83 families. Additionally, its reaction kinetics (*K_m_* = 62 μm; *k*_cat_ = 2.3 s^−1^) were similar to reported values for other enzymes from the GH33 family of bacterial exosialidases (Fig. 4) (45–47).

A key difference between ORF12p and known exosialidases was its mechanism of catalysis. Glycoside hydrolases are grouped into two main mechanistic classes: inverting or retaining enzymes, depending on whether the stereochemistry of the substrate's anomeric carbon is inverted or retained upon hydrolysis. Our proton ^1^H NMR data unambiguously showed that ORF12p liberated β-Neu5Ac as the primary product of hydrolysis of the substrate (4MU-α-Neu5Ac) (Fig. 6), indicating that it naturally functions via an inverting catalytic mechanism. This observation is unprecedented as all WT exosialidases that have been described to date (from prokaryotes, eukaryotes, and viruses) hydrolyze terminal Neu5Ac with retention of its anomeric configuration (24–27). However, Watson *et al.* (48) showed that mutations introduced into the active site of one bacterial sialidase could induce a change in catalytic mechanism from retaining to inverting. The authors presented a model whereby water is able to more easily access the active site and directly acts as a nucleophile. To understand how ORF12p achieves inverting catalysis, a deeper exploration of its reaction mechanism is needed. We are currently attempting crystallization of ORF12p for X-ray structure determination to enable the characterization of the molecular determinants of substrate recognition and catalysis.

In summary, we have identified a novel exosialidase using functional screening of a hot spring metagenomic library in *E. coli*. This enzyme is the first member of the novel CAZy family GH156 and is the first reported WT exosialidase to function via an inverting mechanism. This study also highlights the benefits of using function-based screening to identify unique new carbohydrate hydrolases within interesting ecological niches.

## Experimental procedures

Chemicals and solvents were from Sigma–Aldrich. The substrate X-Neu5Ac was from Sigma–Aldrich. The substrate 4MU-α-Neu5Ac was from Toronto Research Chemicals (North York, Canada). The 2AB fluorescently labeled substrates 3′ and 6′-sialyl-*N*-acetyllactosamine-2AB and a 2AB-labeled di-Neu5Ac-terminated biantennary complex *N*-glycan (termed G2S2-Ac) were from Prozyme (Hayward, CA). The 2AB-labeled di-Neu5Gc-terminated biantennary complex *N*-glycan (termed G2S2-Gc) was from Tokyo Chemical Industry (Tokyo, Japan). All DNA enzymes and enzymes for SMRTbell complex library preparation were from New England Biolabs. MilliQ^TM^ purified water (Millipore Sigma) was used in all experiments.

### Metagenomic DNA library construction

Samples from a hot spring located in Dixie Valley, Nevada, were collected in 2013. Environmental DNA was subsequently extracted and frozen until use. Isolated DNA was concentrated to 548 ng/μl using a 0.45× volume of AMPure PB magnetic beads (Pacific Biosciences, Menlo Park, CA) and used to produce a DNA library using the CopyRight v2.0 Fosmid cloning kit (Lucigen Corporation, Middleton, WI) following the manufacturer's instructions. Briefly, DNA was end-repaired and size-selected using a 1% low melting point agarose gel run overnight at 35 V. DNA fragments from 30–70 kb were extracted from the gel using 1 unit of β-agarase I (New England Biolabs) for each 100 μl of melted agarose. The end-repaired and size-selected DNA was ligated to the pSMART FOS cloning vector. Phage packaging of the resulting clones was performed using Gigapack III XL packaging extracts (Agilent Technologies, Santa Clara, CA). Replicator FOS cells were transfected with the packaging reaction and plated on YT-CXIS agar medium (8 g of Bacto-tryptone, 5 g of yeast extract, 5 g of NaCl, 15 g agar/liter containing 12.5 μg/ml of chloramphenicol, 40 μg/ml 5-bromo-4-chloro-3-indolyl-β-d-galactopyranoside (X-gal), 0.4 mm isopropyl β-d-1-thiogalactopyranoside (IPTG), and 5% (w/v) sucrose) and incubated overnight at 37 °C. A total of 616 colonies were harvested and archived in two 384-well plates in 20% sterile glycerol (v/v).

### DNA library quality assessment

Restriction digestion was used to assess the average insert size in the fosmid DNA library. Fosmids were individually isolated from 12 randomly selected library clones using the FosmidMAX^TM^ DNA purification kit (Lucigen Corporation). Each fosmid was digested with SbfI (New England Biolabs), a unique restriction site in the pSMART FOS vector. Digestion products were separated by electrophoresis on a 1% agarose gel to estimate the average insert size of cloned DNA.

To assess the cloned DNA insert species diversity, the 12 randomly selected clones were subjected to Sanger sequencing using the T7 universal primer or pCC1 forward and reverse sequencing primers (Epicenter, Madison, WI) (Table S1). These primers allowed sequencing of ∼500–800 bp from the insert ends. BLASTN was used to probe the GenBank^TM^ sequence repository for known identical sequences to assist in identification of the species of origin for the cloned DNA fragments.

### Screening for sialidase activity

The metagenomic library was screened for sialidase activity using two different approaches. In an agar plate assay, library clones were spotted onto agar plates (10 g of tryptone, 5 g of yeast extract, 10 g of NaCl, 1 g of dextrose, 1 g of MgCl_2_-6H_2_O, 20 g of bacto-agar, and 2 ml of 2 m NaOH per liter) supplemented with 60 μg/ml X-Neu5Ac dissolved in dimethylformamide using a 384 Slot Pin Multi-Blot^TM^ replicator (V&P Scientific Incorporation, San Diego, CA). After overnight incubation at 37 °C, the plates were stored at 4 °C until blue color developed (several days). For sialidase activity screening in *E. coli* cell lysates, the library clones were grown in 50-μl LB liquid cultures (10 g of tryptone, 5 g of yeast extract, 10 g of NaCl, 1 g dextrose, 1 g of MgCl_2_–6H_2_O, 2 ml of 2 m NaOH per liter, containing chloramphenicol 12.5 μg/ml and 1× inducing solution (Lucigen Corporation)) in 384-well plates and incubated overnight at 37 °C. 50 μl of lysis buffer (Thermo Fisher Scientific) containing 40 μg/ml of the sialidase substrate 4MU-α-Neu5Ac were added to the liquid cultures. The mixtures were incubated overnight at 37 °C in a static incubator. Fluorescence at λ_ex_ = 365 nm and λ_em_ = 445 nm was read with a SpectraMax Plus 384 microplate reader (Molecular Devices, Sunnyvale, CA).

### Pacific Biosciences sequencing of fosmid clones

Fosmid DNA was extracted using the FosmidMAX^TM^ DNA purification kit (Lucigen Corporation) following the manufacturer's instructions. DNA was isolated from 40-ml LB cultures supplemented with 12.5 μg/ml chloramphenicol and 1× inducing solution (Lucigen Corporation). A 10-kb SMRTbell library was prepared as follows. 10 μg of DNA in 150 μl of TE buffer (10 mm Tris, pH 8.0, 0.1 mm EDTA) were sheared using g-tubes (Covaris, Woburn, MA). The g-tubes were centrifuged at 4500 rpm (Eppendorf 5424 centrifuge) for 30 s with the cap up, followed by 30 s with the cap down, three times each for a total of six spins. Sheared DNA was concentrated using 0.45× volume of AMPure PB magnetic beads (Pacific Biosciences). The beads were incubated for 10 min in a ThermoMixer C (Eppendorf, Hamburg, Germany) at 1,400 rpm and then washed twice on a magnetic rack with 500 μl of freshly prepared 70% ethanol. DNA was eluted from the beads by addition of 20 μl of TE buffer and incubation for 2 min in a ThermoMixer C at 1400 rpm 2 μl of PreCR Repair Mix (New England Biolabs) and 5 μl of NEBNext End Repair Reaction Buffer (New England Biolabs) in a 50 μl of total volume reaction was added to repair the damaged DNA template. The reaction was incubated at 37 °C for 20 min. DNA was end-repaired by addition of 2.5 μl of NEBNext End repair mix (New England Biolabs) (incubation at 25 °C for 5 min) and then purified with 0.45× volume of AMPure PB magnetic beads and eluted with 20 μl of TE. DNA was blunt-ligated with 2.5 μl of 80 μm SMRTbell adaptors (Integrated DNA Technologies, San Jose, CA) using 17.5 μl of blunt/TA ligase master mix (New England Biolabs). The ligation reaction was incubated at 25 °C for 15 min. Ligase was removed using the Monarch PCR and DNA cleanup kit (New England Biolabs). Unligated DNA fragments were removed by digestion with exonuclease III and VII (100 units each) in the presence of 4 μl of buffer for T4 DNA ligase with 10 mm ATP. Finally, two AMPure PB magnetic bead purifications were performed. The SMRTbell library was ultimately eluted with 15 μl of TE buffer. MagBead complexes were prepared on the day of sequencing using the binding calculator tool and enzymes from Pacific Biosciences. The final library was sequenced on a PacBio RS II platform using the P6 chemistry and one single-molecule real-time cell for a 360-min movie.

Reads were assembled *de novo* using the RS_HGAP_Assembly.3 protocol (PacBio Portal). ORFs encoded by the assembled contig were predicted using MetaGeneMark (32). The deduced protein sequence of each ORF was subjected to BLASTP analysis against the GenBank^TM^ nonredundant protein database. ORFs that showed good identity and coverage scores to several proteins of the same function were annotated with this putative function. ORFs that showed no significant homology to any protein of known function or that matched proteins annotated as “hypothetical proteins” were assigned as such for our study.

### Tn5 mutagenesis

A library of random mutants of fosmid G7 was generated using the EZ-Tn5^TM^ <Kan-2> insertion kit (Lucigen Corporation) following the manufacturer's instructions. Briefly, an equimolar amount (0.007 pmol) of the EZ-Tn5^TM^ <KAN-2> transposon and target DNA (G7 fosmid) were used to maximize the insertion efficiency while minimizing multiple insertion events. The 10-μl reaction was incubated at 37 °C for 2 h and stopped upon addition of 1 μl of EZ-Tn5 10× stop solution and incubation at 70 °C for 10 min. 1 μl of the reaction was added to 50 μl of thawed electrocompetent cells EC300110 (Lucigen Corporation) in a prechilled tube. Electroporation was performed with a Gene Pulser Xcell^TM^ electroporation system (Bio-Rad) following the manufacturer's instructions (1-mm gap, 1800 V, 25 microfarad, and 200 Ω). SOC medium (New England Biolabs) was added immediately after electroporation (950 μl), and the cells were transferred to a 15-ml tube for incubation at 37 °C with shaking for 1 h. Transformed cells were plated on LB + 12.5 μg/ml chloramphenicol and 50 μg/ml kanamycin. 192 random mutants were arrayed in two 96-well plates. Sialidase activity in both plates was investigated using the lysis-based assay with 4MU-α-Neu5Ac substrate as described above.

### In vitro and in vivo sialidase expression

Sialidase candidates ORF9 and ORF12 were each expressed *in vitro* using the PURExpress *in vitro* protein synthesis kit (New England Biolabs) following the manufacturer's instruction. PURExpress DNA templates were generated by PCR using primers specific to ORF9 or ORF12 (Table S1). PCR was performed using 250 ng of fosmid G7 as template, 0.75 μl of each primer (20 μm), 15 μl of Q5 hot start high-fidelity 2× Master Mix (New England Biolabs) in a 30-μl total reaction volume. Thermocycling consisted of 25 cycles (98 °C for 10 s, and 72 °C for 2 min). The amplified product was purified using the Monarch PCR clean-up kit (New England Biolabs). For *in vitro* protein synthesis, 1 μl of amplified ORF9 or ORF12 DNA was mixed with 10 μl of solution A, 7.5 μl of solution B, and 0.5 μl of RNase inhibitor murine (New England Biolabs) and incubated for 2 h at 37 °C to express the desired protein. Expression was verified by separating 2.5 μl of the reaction on a Novex 10–20% Tris–glycine gel (Thermo Fisher Scientific). Additionally, sialidase activity of *in vitro* expressed proteins was assessed by incubating 20 μl of PURExpress product with 4 μl of 100 μg/ml 4MU-α-Neu5Ac at 37 °C for 1 h and reading fluorescence at λ_ex_ = 365 nm and λ_em_ = 445 nm in a SpectraMax microplate fluorometer.

ORF12 was fused with a hexahistidine tag coding sequence at its 3′ end (for a C-terminal tag) and cloned into the pJS119K vector (49). Primers were designed using the NEBuilder assembly online tool (New England Biolabs) (Table S1). Linearization of the vector was performed by PCR with Q5 hot start high-fidelity 2× Master Mix (New England Biolabs) for 25 cycles (98 °C for 10 s, 60 °C for 30 s, and 72 °C for 2 min). An insert was also prepared by PCR using 25 cycles of 98 °C for 10 s, 60.5 °C for 30 s, and 72 °C for 2 min with Q5 hot start high-fidelity 2× Master Mix. The construct was assembled using the NEBuilder HiFi DNA assembly cloning kit (New England Biolabs) and transfected into New England Biolabs 5-α competent *E. coli* cells following the manufacturer's instructions. The clone was verified by Sanger sequencing and used to transform New England Biolabs Express competent cells. Expression under the Ptac promoter was performed at 18 °C overnight upon addition of IPTG to 0.4 mm. Cells from 1 liter of LB culture were harvested by centrifugation at 15,000 × *g* for 10 min at 4 °C. The cell paste was resuspended in 30 ml of 20 mm sodium phosphate, pH 7.4, 500 mm NaCl, and 20 mm imidazole buffer and lysed using a TS Benchtop Series cell disruptor (Constant Systems Limited, Daventry, UK) at 32 kilo pounds per square inch (kPsi). Expressed protein was purified on a 5-ml His-Trap^TM^ FF column (GE Healthcare). The bound ORF12–His_6_ protein was eluted with 20 mm sodium phosphate, pH 7.4, 500 mm NaCl, and 500 mm imidazole by gradient elution over 20 column volumes (from 0 to 100% of elution buffer). Fractions containing pure protein were pooled and dialyzed against 20 mm sodium phosphate, pH 7.4, containing 500 mm NaCl, 1 mm EDTA.

### Sialidase biochemical characterization

One unit of the ORF12 sialidase was defined as the amount of enzyme required to cleave Neu5Ac from 1 nmol of 4MU-α-Neu5Ac in 1 h at 37 °C in 20 mm sodium phosphate, pH 7.4. The effect of pH and temperature on enzyme activity was investigated by incubating 0.5 unit of enzyme with 1.7 pmol of 3′-sialyl-*N*-acetyllactosamine-2AB for 3 h at 37 °C. These conditions led to incomplete cleavage so that both substrate and product could be monitored. A range of pH from 4.5 to 9.5 (50 mm sodium acetate buffer for pH between 4.5 and 5.5, 20 mm sodium phosphate buffer for pH between 5.5 and 8.0, and 50 mm Tris-HCl buffer for pH between 8.0 and 9.5) at 37 °C and temperature ranges from 15 to 70 °C in 50 mm sodium acetate, pH 5.0, were tested. The effect of metal ions was tested by incubating 0.5 unit of enzyme with 1.7 pmol of 3′-sialyl-*N*-acetyllactosamine-2AB in 50 mm sodium acetate, pH 5.0, with 5 mm of NiSO_4_, CaCl_2_, MnSO_4_, MgCl_2_, FeSO_4_, ZnCl_2_, or CuSO_4_, respectively, for 3 h at 37 °C. After incubation, the reactions were dried by vacuum evaporation and resuspended in 1.8 μl of water and 13.2 μl of acetonitrile for a 12:88 ratio. 12 μl were injected into a Waters Acquity BEH glycan amide column (2.1 × 150 mm, 1.7 μm) on a Waters Acquity UPLC H-Class instrument (Waters Corporation, Milford, MA) equipped with a quaternary solvent manager and a fluorescence detector. 50 mm ammonium formate buffer, pH 4.4, and 100% acetonitrile were used, respectively, as solvent A and B. The gradient used was 0–1.5 min, 12% solvent A; 1.5–35 min, 47% solvent A; 35–36 min, 70% solvent A; and 36.5–42 min, 12% solvent A with a flow rate of 0.561 ml/min. The samples were kept at 5 °C prior to injection, and separation was performed at 30 °C. The fluorescence detection wavelengths were λex = 330 nm and λem = 420 nm with a data collection rate of 20 Hz. The quantity of uncleaved substrate and released product were analyzed with Empower 3 chromatography work station software (Waters Corporation). Peak areas were quantitated by integration, and relative activity was calculated.

Sialidase specificity was assessed by testing its efficacy on 2AB-labeled 3′ and 6′-sialyl-*N*-acetyllactosamine substrates (termed 3′-SLN-2AB and 6′-SLN-2AB, respectively), G2S2-Ac, G2S2-Gc, and the GD3 ganglioside-released glycan (Neu5Ac(α2–8)Neu5Ac(α2–3)Gal(β1–4)Glc-2AB). The released glycan headgroup substrate from GD3 ganglioside was prepared as follows: glycans from 10 nmol of GD3 ganglioside dissolved in methanol were released with 20 milliunits of endoglycoceramidase I (New England Biolabs) in a 10-μl reaction and incubated for 24h at 37 °C. The reaction mix was passed through a Nanosep 10K Omega centrifugal device (Pall Corporation, Westborough, MA) and centrifuged at 12,000 rpm for 4 min to remove the enzyme. The filtrate was dried in a vacuum evaporator prior to 2AB labeling for 2 h at 65 °C with 10 μl of 2AB labeling mix (350 mm 2AB, 1 m sodium cyanoborohydride in 7:3 DMSO:acetic acid). Labeled glycans were cleaned-up with a HILIC detergent removal Microspin cartridge (The Nest Group Inc., Southborough, MA).

Enzyme (1 or 10 units) was mixed with each of the substrates in 20-μl reactions in 50 mm sodium acetate, pH 5.0, and incubated at 37 °C overnight. Negative and positive control experiments were performed in the same conditions with no enzyme and 1 μl of NeuA (New England Biolabs), respectively. Reactions were dried in a vacuum evaporator and analyzed by UPLC–HILIC–FLR as described above. ORF12p–His activity was also tested on a fetuin *O*-glycan library purchased from Ludger (Oxfordshire, UK) and 2AB-labeled as described above.

### Reaction kinetics

To determine the kinetic parameters of ORF12p–His, 20 units of ORF12p–His was incubated at 37 °C with different concentrations of 4MU-α-Neu5Ac (60–280 μm) in 50 mm sodium acetate, pH 5.0. Each substrate concentration was tested in triplicate. Aliquots (50 μl) of each reaction were removed at different time points over a 5-min incubation. They were immediately mixed with 50 μl of 1 m sodium carbonate (pH 10.9) to stop the reaction. Fluorescence was read at λ_ex_ = 365 nm and λ_em_ = 445 nm in a SpectraMax microplate fluorometer.

### NMR spectroscopy

NMR was performed by the Complex Carbohydrate Research Centre (Athens, Georgia). A solution was prepared in a 5-mm NMR tube with 320 μl of 1.78 mg/ml 4MU-α-NeuAc in D_2_O (2 mm final concentration after addition of enzyme), 60 μl of 200 mm sodium phosphate in D_2_O, pH 7.4 (20 mm final concentration after addition of enzyme), and 190 μl of D_2_O. The solution was mixed, and the NMR tube placed into a 600-MHz Inova NMR spectrometer (Agilent Technologies, Santa Clara, CA) at 25 °C. A one-dimensional proton NMR experiment with water presaturation (using the two-step purge option) was recorded as the *t* = 0 measurement. Then 30 units of ORF12p–His sialidase or 240 units of NeuA (New England Biolabs) was added, the NMR tube was inverted several times to ensure good mixing, and the tube was replaced into the NMR spectrometer. One-dimensional proton spectra with water presaturation were recorded at time intervals with 32 transients each. Chemical shifts were referenced relative to the residual HDO peak, set at 4.78 ppm.

### Armatimonadetes homolog expression

The gene encoding the hypothetical protein OIO94155 from *Armatimonadetes* was synthesized by GenScript (Piscataway, NJ) in pUC57. The gene was subcloned into pJS119K using the HiFi DNA assembly cloning kit (New England Biolabs) with primers designed with NEBuilder assembly online tool (Table S1). The clone was verified by Sanger sequencing and used to transform New England Biolabs Express competent cells. Expression under the Ptac promoter was performed at 30 °C for 4 h upon addition of IPTG to 0.4 mm. Crude lysate was prepared by sonication and assayed with 4MU-α-Neu5Ac by mixing 5 μl of crude lysate with 4 μl of 100 μg/ml 4MU-α-Neu5Ac and 15 μl of 20 mm MES buffer, pH 6.5. After 1 h of incubation at 37 °C, the fluorescence of the reaction was read at λ_ex_ = 365 nm and λ_em_ = 445 nm in a SpectraMax microplate fluorometer. Lysate from New England Biolabs Express cells containing the empty pJS119K vector and lysate from the pJS119K-ORF12p construct were prepared and assayed in the same conditions to serve as negative and positive controls, respectively.

## Author contributions

L. C., M. B. G., and B. H. data curation; L. C., M. B. G., and B. H. formal analysis; L. C. and M. B. G. methodology; L. C. and C. H. T. writing-original draft; L. C., M. B. G., E.R., B. H., and C. H. T. writing-review and editing; M. B. G. validation; E. R. and C. H. T. supervision; C. H. T. conceptualization; C. H. T. funding acquisition; C. H. T. investigation; C. H. T. project administration.

## Supplementary Material

Supporting Information

## Footnotes

2 Please note that the JBC is not responsible for the long-term archiving and maintenance of this site or any other third party hosted site.

## Footnotes

3 The abbreviations used are:

X-Neu5Ac: 5-bromo-4-chloro-3-indolyl-α-d-*N*-acetylneuraminic acid
4MU-α-Neu5Ac: 2′-(4-methylumbelliferyl)-α-d-*N*-acetylneuraminic acid
contig: contiguous sequence obtained by assembly of shorter overlapping sequences
2AB: 2-aminobenzamide
SLN-2AB: 2AB-labeled sialyl-*N*-acetyllactosamine
UPLC–HILIC–FLR: ultra-HPLC with fluorescence detection
NeuA: α2–3,6,8,9-neuraminidase A
IPTG: isopropyl β-d-1-thiogalactopyranoside.
