# Supplementary material for: Functional metagenomics identifies an exosialidase with an inverting catalytic mechanism that defines a new glycoside hydrolase family (GH156)
Source: J Biol Chem. 2018 Sep 24;293(47):18138–50. doi: 10.1074/jbc.RA118.003302 (PMC6254351; doi:10.1074/jbc.RA118.003302)
Supplement: Supporting Information [file supp_293_47_18138__index.html]

Functional metagenomics identifies an exosialidase with an inverting catalytic mechanism that defines a new glycoside hydrolase family (GH156) — A new family of exosialidases with an inverting mechanism — Functional metagenomics identifies an exosialidase with an inverting catalytic mechanism that defines a new glycoside hydrolase family (GH156) — A new family of exosialidases with an inverting mechanism — Supporting Information 

# Functional metagenomics identifies an exosialidase with an inverting catalytic mechanism that defines a new glycoside hydrolase family (GH156)

## Supporting Information

- Supporting information for: Functional metagenomics identifies an exosialidase with an inverting catalytic mechanism that defines a new glycoside hydrolase family (GH156) - Supporting data presented in the main text.
