# Supplementary material for: Functional metagenomics identifies an exosialidase with an inverting catalytic mechanism that defines a new glycoside hydrolase family (GH156)
Source: J Biol Chem. 2018 Sep 24;293(47):18138–50. doi: 10.1074/jbc.RA118.003302 (PMC6254351; doi:10.1074/jbc.RA118.003302)
Supplement: Supporting Information [file supp_RA118.003302_137217_2_supp_200622_p4w49w.pdf]

Functional metagenomics identifies an exosialidase with an inverting catalytic mechanism that defines a new glycoside hydrolase family (GH156)

Léa Chuzel[1,2], Mehul B. Ganatra[1], Erdmann Rapp[2,3], Bernard Henrissat[4,5,6] and Christopher H. Taron[1]*

From [1]New England Biolabs, 240 County Road, Ipswich, MA 01938, USA; [2]Max Planck Institute for Dynamics of Complex Technical Systems, Sandtorstrasse 1, 39106 Magdeburg, Germany; [3]glyXera GmbH, Leipziger Straße 44, 39120 Magdeburg, Germany; [4]Architecture et Fonction des Macromolécules Biologiques, CNRS, Aix-Marseille Université, F-13288 Marseille, France; [5]INRA, USC 1408 AFMB, 13288, Marseille, France; [6]Department of Biological Sciences, King Abdulaziz University, Jeddah, 21589, Saudi Arabia

This document provides information supplemental to the main text:

Table S1. Nucleotide primers used in this study

Figure S1. Screening for sialidase activity of G7 fosmid mutants

Figure S2. ORF12p activity on a fetuin 2AB-labelled *O*-glycan library

Table S1. Nucleotide primers used in this study

Primer name	Primer sequence 5' –> 3'	Use
T7 universal primer	TAATACGACTCACTATAGGG	Sanger sequencing primers for assessment of metagenomic library diversity
pCC1 Forward primer	GGATGTGCTGCAAGGCGATTAAGTTGG	
pCC1 Reverse primer	CTCGTATGTTGTGTGGAATTGTGAGC	
ORF12 PURExpress forward primer[1]	GCGAATTAATACGACTCACTATAGGGC TTAAGTATAAGGAGGAAAAAATATGA GGCCGGAGACAATACCGGGGATC	PCR primers for generation of an ORF12 template for *in vitro* transcription/translation
ORF12 PURExpress reverse primer[2]	AAACCCCTCCGTTTAGAGAGGGGTTAT GCTAGTCAGGAGTGCCAGGGGCGTAT GAGAAA	
ORF12-6His HiFi forward primer	TAAGCTTAGGAGGTTAACATATGAGGC CGGAGACAATACC	Primers for cloning of histidine tagged ORF12 into the expression vector pJS119K. Designed using NEBuilder assembly tool[3]
ORF12-6His HiFi reverse primer	TCAGTGATGGTGATGGTGATGGGAGTG CCAGGGGCGTAT	
pJS119K forward primer	CATCACCATCACCATCACTGAGAATTC AGCTTGGCTGTTTTG	
pJS119K reverse primer	ATGTTAACCTCCTAAGCTTAATTC	
OIO94155 *Armatimonadetes* forward primer	TAA GCT TAG GAG GTT AAC ATA TGA AGG GTC CGA TCT TCA AC	Primers for subcloning OIO94155 *Armatimonadetes* protein from pUC57 to the expression vector pJS119K. Designed using NEBuilder assembly tool[3]
OIO94155 *Armatimonadetes* reverse primer	AAA ACA GCC AAG CTG AAT TCT TAG CCG CTT TTC GCC AC	

[1]Contains a T7 promoter and a ribosome binding site upstream the starting site of the ORF12 coding

[2]Contains a T7 terminator downstream from the stop codon

[3]http://nebuilder.neb.com/

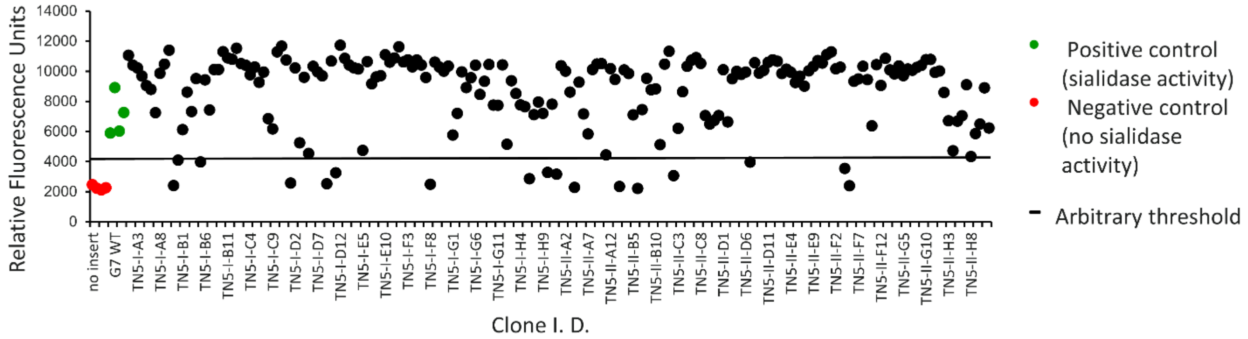

Figure S1. Screening for sialidase activity of G7 fosmid mutants

A library of G7 fosmid mutants, each containing a kanamycin cassette randomly inserted by the Tn5 transposase, was created. The library of mutants was assayed for sialidase activity using 4MU-α-Neu5Ac. Four G7 wild-type clones were also assayed as a positive activity control (green dots) and four no insert fosmid clones (red dots) as a negative activity control. An arbitrary threshold was set to estimate clones with abolished sialidase activity.

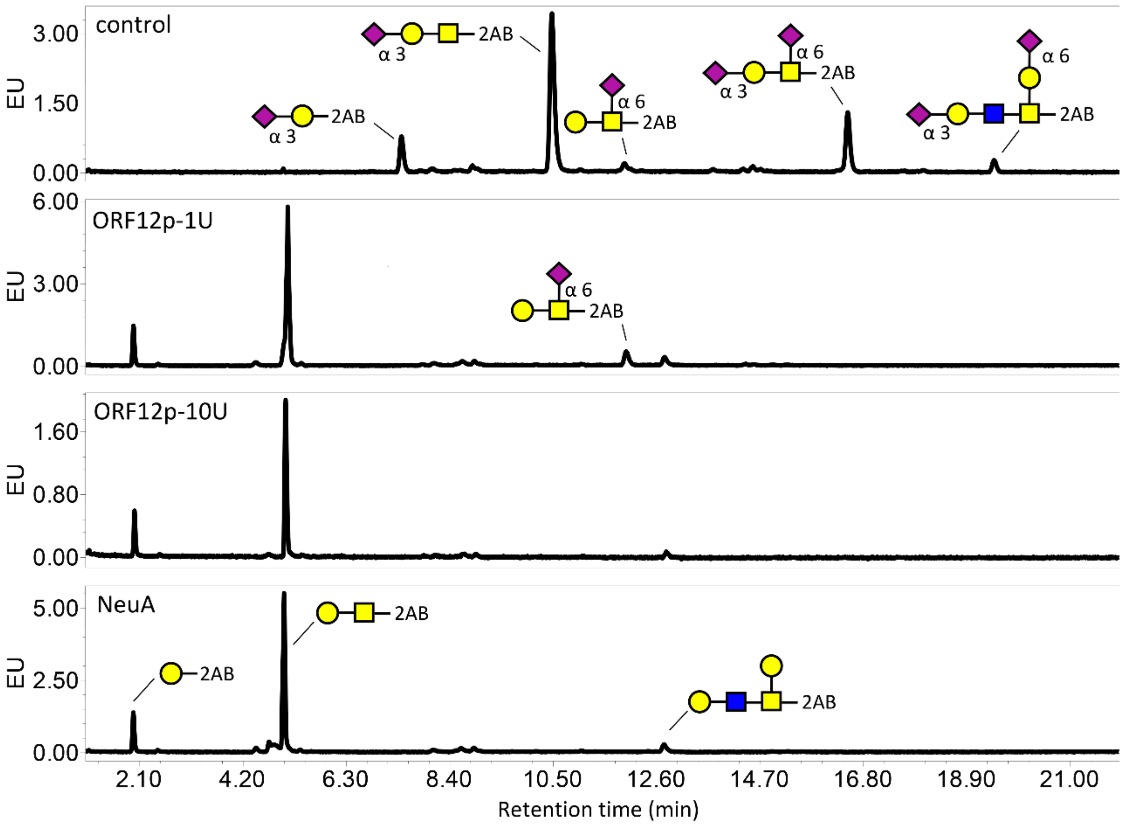

Figure S2. ORF12p activity on a fetuin 2AB-labelled *O*-glycan library.

The ability of ORF12p to cleave 3' or 6' terminal sialic acid linked to galactose or *N*-acetylgalactosamine residue was monitored by HILIC-UPLC-FLR. A library of undigested 2AB-labelled *O*-glycan from bovine fetuin showed 5 major structures at ~7.4, 10.5, 12, 16.5 and 19.5 min retention time (top panel). Neuraminidase A (NeuA) treatment shifted all 5 substrate peaks to 3 peaks at ~2, 5 and 12.8 min retention time (bottom panel). Incubation of the substrates with 10 U of purified ORF12 sialidase resulted in the same shifts (middle panel). Symbolic representation of glycan structures was drawn following the guidelines of the Consortium for Functional Glycomics (48). Abbreviation: EU, emission units.
